# Counterregulatory hormone and symptom responses to hypoglycaemia in people with type 1 diabetes, insulin-treated type 2 diabetes or without diabetes: the Hypo-RESOLVE hypoglycaemic clamp study

**DOI:** 10.1007/s00592-024-02239-8

**Published:** 2024-02-20

**Authors:** Therese W. Fabricius, Clementine E. M. Verhulst, Peter L. Kristensen, Jens J. Holst, Cees J. Tack, Rory J. McCrimmon, Simon R. Heller, Mark L. Evans, Bastiaan E. de Galan, Ulrik Pedersen-Bjergaard, the Hypo-RESOLVE consortium

**Affiliations:** 1 Department of Endocrinology and Nephrology, Nordsjællands Hospital, Hillerød, Denmark; 2 Department of Internal Medicine, Radboud University Medical Centre, Nijmegen, The Netherlands; 3 Department of Biomedical Sciences, Faculty of Health and Medical Sciences, University of Copenhagen, Copenhagen, Denmark; 4 Faculty of Health and Medical Sciences, Novo Nordisk Foundation Center for Basic Metabolic Research, University of Copenhagen, Copenhagen, Denmark; 5 Systems Medicine, School of Medicine, University of Dundee, Dundee, UK; 6 Department of Oncology and Metabolism, University of Sheffield, Sheffield, UK; 7 Welcome MRC Institute of Metabolic Science, University of Cambridge, Cambridge, UK; 8 Department of Internal Medicine, Maastricht UMC+, Maastricht, The Netherlands; 9 CARIM School for Cardiovascular Diseases, Maastricht University, Maastricht, The Netherlands; 10 Department of Clinical Medicine, Faculty of Health and Medical Sciences, University of Copenhagen, Copenhagen, Denmark

**Keywords:** Diabetes, Hypoglycaemia, Hyperinsulinaemic–hypoglycaemic clamp, Counterregulatory response

## Abstract

**Aim:**

The sympathetic nervous and hormonal counterregulatory responses to hypoglycaemia differ between people with type 1 and type 2 diabetes and may change along the course of diabetes, but have not been directly compared. We aimed to compare counterregulatory hormone and symptom responses to hypoglycaemia between people with type 1 diabetes, insulin-treated type 2 diabetes and controls without diabetes, using a standardised hyperinsulinaemic-hypoglycaemic clamp.

**Materials:**

We included 47 people with type 1 diabetes, 15 with insulin-treated type 2 diabetes, and 32 controls without diabetes. Controls were matched according to age and sex to the people with type 1 diabetes or with type 2 diabetes. All participants underwent a hyperinsulinaemic–euglycaemic-(5.2 ± 0.4 mmol/L)-hypoglycaemic-(2.8 ± 0.13 mmol/L)-clamp.

**Results:**

The glucagon response was lower in people with type 1 diabetes (9.4 ± 0.8 pmol/L, 8.0 [7.0–10.0]) compared to type 2 diabetes (23.7 ± 3.7 pmol/L, 18.0 [12.0–28.0], *p* < 0.001) and controls (30.6 ± 4.7, 25.5 [17.8–35.8] pmol/L, *p* < 0.001). The adrenaline response was lower in type 1 diabetes (1.7 ± 0.2, 1.6 [1.3–5.2] nmol/L) compared to type 2 diabetes (3.4 ± 0.7, 2.6 [1.3–5.2] nmol/L, *p* = 0.001) and controls (2.7 ± 0.4, 2.8 [1.4–3.9] nmol/L, *p* = 0.012). Growth hormone was lower in people with type 2 diabetes than in type 1 diabetes, at baseline (3.4 ± 1.6 vs 7.7 ± 1.3 mU/L, *p* = 0.042) and during hypoglycaemia (24.7 ± 7.1 vs 62.4 ± 5.8 mU/L, *p* = 0.001). People with 1 diabetes had lower overall symptom responses than people with type 2 diabetes (45.3 ± 2.7 vs 58.7 ± 6.4, *p* = 0.018), driven by a lower neuroglycopenic score (27.4 ± 1.8 vs 36.7 ± 4.2, *p* = 0.012).

**Conclusion:**

Acute counterregulatory hormone and symptom responses to experimental hypoglycaemia are lower in people with type 1 diabetes than in those with long-standing insulin-treated type 2 diabetes and controls.

**Supplementary Information:**

The online version contains supplementary material available at 10.1007/s00592-024-02239-8.

## Introduction

Despite a century of development and refinement of insulin therapy, hypoglycaemia remains the most common complication, affecting virtually all people with type 1 diabetes and most of those with insulin-treated type 2 diabetes [1]. Thus, people with diabetes depend on physiological and behavioural defence mechanisms to prevent mild hypoglycaemia from progressing to (more) severe episodes [2]. However, the counterregulatory hormone and symptom responses to hypoglycaemia have been observed to wane over time in people with type 1 diabetes and people with longstanding insulin-treated type 2 diabetes [3–6], and evidence suggests that long duration of diabetes increases the risk of severe hypoglycaemia [7–9].

Several studies using the hyperinsulinaemic hypoglycaemic clamp technique have demonstrated that people with type 1 diabetes have less pronounced or absent counterregulatory (hormone) responses, and that these are elicited at lower glucose levels (higher thresholds) compared to controls without diabetes [10]. Exposure to recurrent hypoglycaemia has been shown to suppress counterregulatory responses to subsequent hypoglycaemia, ultimately leading to impaired awareness of hypoglycaemia (IAH), a condition associated with a more than sixfold increased risk of severe hypoglycaemia, with a prevalence of 25% in people with type 1 diabetes [3, 11–15]. In type 2 diabetes, counterregulatory responses have both been reported to be unaffected [16–18] or reduced [6], with counterregulatory failure being described in those with reduced beta-cell function in longstanding disease [6] and the prevalence of IAH averaging 10% in those using insulin [19]. Also, age can significantly impact the response to hypoglycaemia, as it has been reported that the counterregulatory hormone and symptom response to hypoglycaemia in elderly people are activated at lower plasma glucose levels than in younger people [20, 21].

To date, counterregulatory hormone and symptom responses to hypoglycaemia have not been directly compared between people with type 1 diabetes and those with insulin-treated type 2 diabetes vis-à-vis controls without diabetes. This is important since the comparison of the responses to hypoglycaemia between type 1 and type 2 diabetes across different studies is hampered by the highly different experimental protocols that are usually used to induce hypoglycaemia, including the nadir and duration of the hypoglycaemic phase [22]. Therefore, we aimed to compare hormonal and symptom responses to hypoglycaemia in people without diabetes and people with type 1 diabetes and insulin-treated type 2 diabetes, using a similar clamp protocol.

## Materials and methods

### Study design

This two-centre intervention study was performed as part of the Hypo-RESOLVE project [23]. The study was conducted at the Department of Endocrinology and Nephrology at Nordsjællands Hospital Hillerød, Denmark, and the Department of Internal Medicine at Radboud University Medical Centre, Nijmegen, The Netherlands. The study was approved by ethics committees in both countries (H-19005936 and NL67229.091.18) and performed according to the principles of the Declaration of Helsinki. The study was registered at clinicaltrials.gov with the number NCT03976271 and ran from August 2019 until March 2021.

### Study population

We recruited people with type 1 diabetes, insulin-treated type 2 diabetes and two age- and sex-matched control groups without diabetes. People with diabetes were recruited through the diabetes outpatient clinics. We recruited the controls without diabetes using local newspapers and social media advertisements. The body mass index (BMI) varied between 19 and 40 kg/m^2^, age was 18–80 years and blood pressure < 140/90 mmHg. People with diabetes needed to be on a basal-bolus insulin regimen for at least one year and to have a duration of diabetes > 1 year. The main exclusion criteria were HbA_1c_ above 100 mmol/mol (11.3%), use of anti-depressant drugs and severe medical or psychiatric disorders potentially interfering with the perception of hypoglycaemia, and a history of cardiovascular disease (e.g. myocardial infarction, stroke, heart failure or symptomatic peripheral arterial disease) in the past five years before the screening. Pregnancy, breastfeeding or taking no measures for birth control were exclusion criteria for women with child-bearing potential. Participants with diabetes completed Clarke, Gold and Pedersen-Bjergaard questionaries for assessment of awareness of hypoglycaemia [12, 14, 24]. A participant was classified as having IAH when results of at least two of the questionnaires was consistent with IAH. A complete list of inclusion and exclusion criteria and cut-offs for IAH, can be found in the ESM methods.

### Study protocol

People with diabetes were provided with an open intermittently scanned Continuous Glucose Monitoring (isCGM) device (FreeStyle Libre 1®) to record glucose profiles and to avoid hypoglycaemia (< 3.0 mmol/L) 24 h before the experimental day. We rescheduled the experimental day in case of a hypoglycaemic event (< 3.0 mmol/L). We instructed participants on multiple daily injections (MDI) to reduce their basal insulin dose by 25% the night before the clamp and to omit their morning insulin dose. Participants using an insulin pump were asked to turn off the pump 1 h before arriving at the hospital. They were asked to abstain from caffeine-containing substances (e.g. coffee and tea), alcohol and tobacco for at least 24 h and from strenuous exercise 48 h before the clamp.

Participants arrived at the research unit between 07:00 and 08:00 A.M after an overnight fast. A catheter was placed in an antecubital vein in the dominant arm for constant insulin infusion (Novo Rapid®, Novo Nordisk, Bagsværd, Denmark) at 1.5 mU kg^−1^ min^−1^ and a variable infusion of 20% glucose (Baxter B.V., Deerfield, IL or Fresenius Kabi A.B, Sweden), with a minimum of 15 min from cannulation before the beginning of the clamp. To overcome insulin resistance, people with type 2 diabetes and their control group received a higher insulin infusion rate of 3.0 mU kg^−1^ min^−1^[25]. To examine for a potential effect of the higher insulin dose, six controls without diabetes (matched with type 2 diabetes) underwent the experiments twice, once with insulin infusion of 1.5 mU kg^−1^ min^−1^ and another time at 3.0 mU kg^−1^ min^−1^. Another catheter was placed in a retrograde direction in the contralateral hand, which was placed in a heated box (∼55 °C) to arterialise venous blood. Plasma glucose levels were measured at 5- to 10-min intervals using the Biosen-C line glucose analyser (Biosen C-Line; EKF Diagnostics, Cardiff, UK). Baseline plasma glucose levels were measured, whereafter the clamp was started aiming for a euglycaemic level between 5.0 and 5.5 mmol/L for 30 min. If the participants had hyperglycaemia, only the insulin infusion was started until euglycaemia was reached. Following the euglycaemic phase, the plasma glucose level was allowed to decrease to a level of approximately 2.8 mmol/L, and kept there for 60 min. Then, the clamp was terminated, insulin infusion was stopped, and the plasma glucose level was raised to euglycaemia.

### Measurements

Blood samples were drawn at baseline (before the beginning of insulin infusion) and at the end of the hypoglycaemic phase for measurements of adrenaline, noradrenaline, cortisol, and growth hormone. Glucagon levels were assessed at the beginning of the euglycaemic phase and the end of the hypoglycaemic phase. We also sampled blood for measurement of inflammatory markers [26], performed echocardiography, and applied cognitive function tests [27], data which are or will be published elsewhere. We assessed symptoms using the validated modified Edinburgh Hypoglycaemia Score, using a 7-point scale with symptoms ranked from 1 (none) to 7 (severe) [28]. The symptoms were divided into autonomic symptoms (sweating, anxiety, tingling of hands and feet, palpitations, hunger, trembling and shivers/tremor), neuroglycopenic symptoms (feeling warm, confused, inability to concentrate, blurry vision, tiredness, difficulty of speaking, weakness, double vision, dizziness, drowsiness) and general symptoms (headache and nausea).

### Laboratory analysis

HbA_1c_ was assessed by the TOSOH G8 and G11 HPLC-analyser (Sysmex). Plasma C-peptide was measured by R&D Duoset ELISA Human C-peptide DY962505. Plasma adrenaline and noradrenaline were measured by high-performance liquid chromatography in combination with fluorometric detection. Plasma insulin was analysed with an in-house radioimmunoassay, which measured endo- and exogenous insulin. Plasma glucagon was measured with a radioimmunoassay using a C-terminal glucagon-specific antibody (code no 4305) [29]. Plasma cortisol and growth hormone were determined by a routine analysis method with an electrochemiluminescent immunoassay on a Modular Analytics E170 (Roche Diagnostics, GmbH, Germany).

### Statistics

All normally distributed data are shown as mean ± SD and non-normally distributed data as median [Interquartile range] ([IQR]). Counterregulatory hormones and symptom scores are displayed with mean ± SE regardless of distribution, and non-normally data are also displayed with median [IQR]. Baseline variables were compared using independent samples *t*-test. Multiple linear regression analyses were used to compare the effect of hypoglycaemia on counterregulatory hormones and symptoms between groups, with baseline values and groups as covariates. The following comparisons were made throughout the article to examine differences between the groups: Type 1 diabetes versus matched controls without diabetes, type 2 diabetes versus matched controls without diabetes, and type 1 versus type 2 diabetes. A sensitivity analysis based on propensity score was performed to compare participants with type 1 diabetes and matched controls, available in electronic supplementary material (ESM). The level of statistical significance was set at 5% (two-sided). IBM SPSS Statistical software, version 25.0 (IBM, Armonk, NY), was used for analysis.

## Results

A total of 94 people were included in the study. Controls for people with type 1 diabetes were well-matched for age and sex to the type 1 diabetes subgroup, although the BMI was higher in the latter (*p* < 0.001) (Table 1). People with type 2 diabetes and their controls without diabetes were well-matched on all three parameters. As expected, people with type 2 diabetes were older, had a higher BMI, and had a shorter duration of diabetes than people with type 1 diabetes. All participants with type 2 diabetes had preserved endogenous insulin secretion with C-peptide levels of 1.35 [0.47–2.99] nmol/L. Thirty-two of the included people with type 1 diabetes regularly used Continuous Glucose Monitoring (CGM) or isCGM for glucose monitoring, and 21 participants used an insulin pump (Table 1). Among people with type 2 diabetes, only one used isCGM and an insulin pump.

**Table 1:** Participant characteristics

		Type 1 diabetes	Type 2 diabetes	Type 1 controls without diabetes	Type 2 controls without diabetes
Participants, *n*		47	15	16	16
Gender (M/F)		23/24	9/6	7/9	9/7
Age, years		50.0 [28.0–63.0]	62.0 [55.0–68.0]*	47.5 [24.5–64.5]	57.0 [52.3–61.8]
Duration of diabetes, years		21.0 [10.0–35.0]	14.0 [10.0–20.0]*	–	–
Duration of insulin treatment, years		20.7 [9.8–34.6]	2.9 [1.1–15.9]**		
HbA_1c_ % (mmol/mol)		7.8 ± 3.1 (61.6 ± 9.9)	8.0 ± 3.2 (63.5 ± 11.2)	5.2 ± 2.5 (33.6 ± 3.5)*	5.4 ± 2.2 (35.6 ± 2.2)**
%		7.8 ± 0.9	8.0 ± 1.0	5.2 ± 0.3*	5.4 ± 0.2**
C-peptide nmol/L		< 0.47 [< 0.47- < 0.47]	1.35 [0.47–2.99]	–	–
Awareness, Normal awareness/Impaired awareness		21/26	13/2	–	–
BMI, kg/m^2^		26.4 ± 3.6	29.0 ± 4.3*	22.6 ± 2.8*	28.0 ± 4.4
Diabetes complications	Retinopathy, *n* (%)	12 (25.5)	2 (13.3)	–	–
	Neuropathy, *n* (%)	9 (19.1)	2 (13.3)	–	–
	Nephropathy, *n* (%)	1 (2.1)	1 (6.7)	–	–
Insulin treatment other glucose lowering medication	CSII, *n* (%)	21 (44.7)	1 (6.7)	–	–
	Injection, *n* (%)	26 (55.3)	14 (93.3)	–	–
	Oral, *n* (%)	0 (0.0)	10 (66.7)	–	–
	SGLT2 inhibitor	–	1 (6.7)	–	–
	Metformin	–	10 (66.7)	–	–
	GLP-1-receptor agonist	–	2 (13.3)	–	–
	Sulfonylurea	–	3 (20.0)	–	–
	Insulin dose, IU/day	50.1 ± 23.5	71.7 ± 54.6	–	–
CGM	*n* (%)	13 (27.7)	–	–	–
isCGM	*n* (%), (with alarm, *n* (%))	19 (40.4), (2 (4.3))	1 (6.7), (1 (6.7))	–	–

Data are n (%), mean ± SD or median [IQR]. *CSII* Continuous Subcutaneous Insulin Infusio, *MDI:* Multiple Daily Injections*. SGLT-2-inhibitor:* Sodium-glucose- co-transporter 2, *GLP-1- receptor agonist:* Glucagon Like Peptide-1 receptor agonists*, CGM* Continuous Glucose Monitoring*, IsCGM* Intermittently Scanned Continuous Glucose Monitoring device (FreeStyle Libre®). *p < 0.05 vs type 1 diabetes,**p < *0.005 vs type 2 diabetes*

### Plasma glucose values

**Fig. 1:**
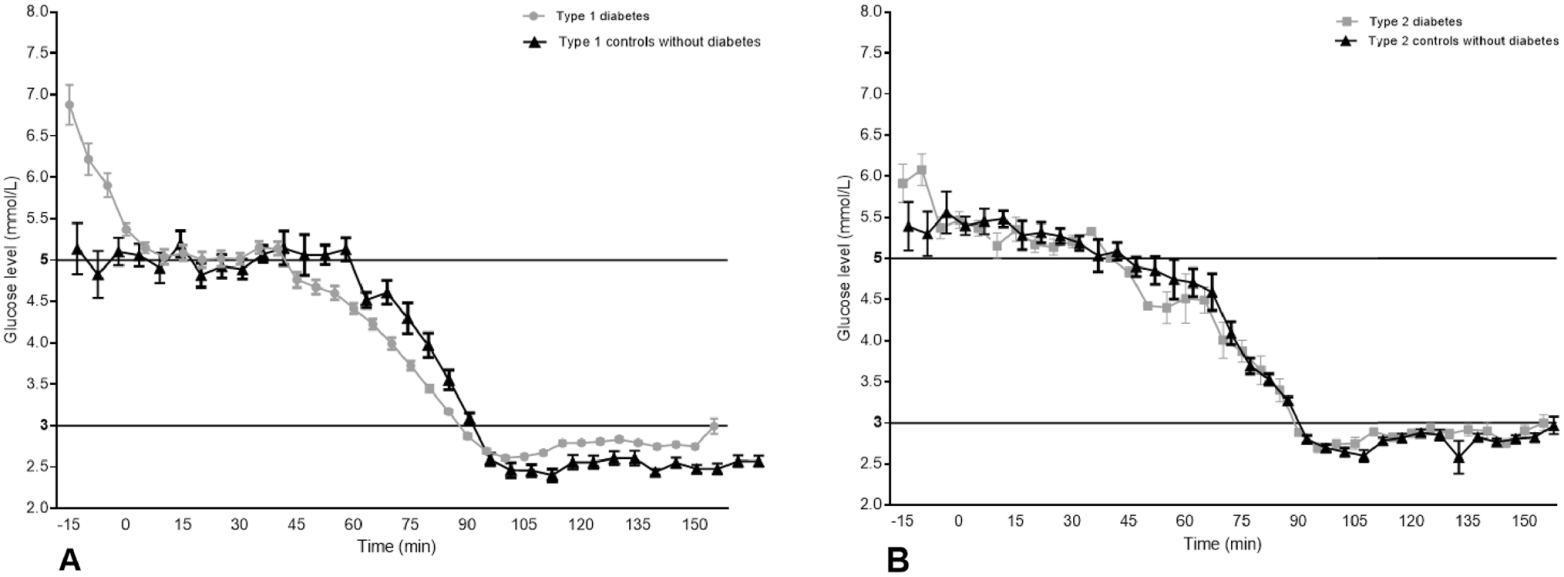
Glucose levels during the clamps. Data are means ± SE. Participants (47 with type 1 diabetes, 15 with type 2 diabetes, and two matched control groups (*n* = 16)), were held in euglycaemia (5.0–5.5 mmol/L) for 30 min, and at hypoglycaemia (2.8 mmol/L) for 60 min

The mean plasma glucose levels during the clamp for all four groups are shown in Fig. 1. Baseline plasma glucose values were higher in the type 1 diabetes group (11.7 ± 3.6 vs 5.7 ± 0.5 mmol/L, *p* < 0.005) and in the type 2 diabetes group (9.6 ± 4.7 vs 5.9 ± 0.5 mmol/L, *p* < 0.005), when compared to the two control groups. The duration for reaching euglycaemia level was 43 ± 3 min for the type 1 diabetes group, 40 ± 5 min for the group with type 2 diabetes and 5 ± 3 and 5 ± 2 min for the healthy controls to type 1 and type 2 diabetes, respectively. Under clamped euglycaemic conditions, plasma glucose levels were similar across the groups (5.2 ± 0.4 mmol/L), with a mean coefficient of variation (CV) of 5.9 ± 2.8%. During the hypoglycaemic phase, the mean plasma glucose level was 2.75 ± 0.95 vs 2.85 ± 0.14 for participants with type 1 and type 2 diabetes, *p* = 0.002. The plasma glucose level was lower in the groups with type 1 diabetes and higher in the group with type 2 diabetes when compared to their matched controls groups (2.85 ± 0.19, *p* = 0.013 and 2.75 ± 0.06, *p* = 0.019), with an overall CV of 6.4 ± 2.6%.

### Insulin concentration

At baseline, insulin levels were higher in people with type 2 diabetes (250 ± 74 mE/L) when compared to their matched control group (23 ± 9 mE/L, *p* = 0.002) and to people with type 1 diabetes (114 ± 19 mE/L, *p* = 0.005), who also had higher values than their corresponding control group (17 ± 6 mE/L, *p* = 0.002). Under clamped conditions, the insulin concentration was more than twice as high in the group with type 2 compared to the group with type 1 diabetes. Insulin levels were higher in people with type 1 diabetes, as compared to the control group without diabetes (214 ± 22 mE/L vs 102 ± 11 mE/L, *p* = 0.003). Similarly, insulin levels were higher in the type 2 diabetes group than in controls without diabetes (546 ± 71 mE/L vs 347 ± 40 mE/L, *p* = 0.019).

### Glucose infusion rate (GIR)

In the normoglycaemic phase, GIR averaged 4.3 ± 2.0 and 7.2 ± 2.0 mg kg^−1^ min^−1^ in the group with type 1 diabetes and controls without diabetes, respectively (*p* < 0.001). GIR was significantly lower in the group with type 2 diabetes compared to matched controls (3.7 ± 2.0 and 5.6 ± 2.6 mg kg^−1^ min^−1^, *p* < 0.001). In the hypoglycaemic phase, GIR averaged 3.1 ± 1.6 and 3.8 ± 2.4 mg kg^−1^ min^−1^ in groups with type 1 diabetes and matched controls (*p* < 0.001), GIR was significantly lower in the group with type 2 diabetes compared to matched controls (1.8 ± 1.2 and 3.9 ± 1.3 mg kg^−1^ min^−1^, *p* < 0.001).

### Counterregulatory hormone responses

**Fig. 2:**
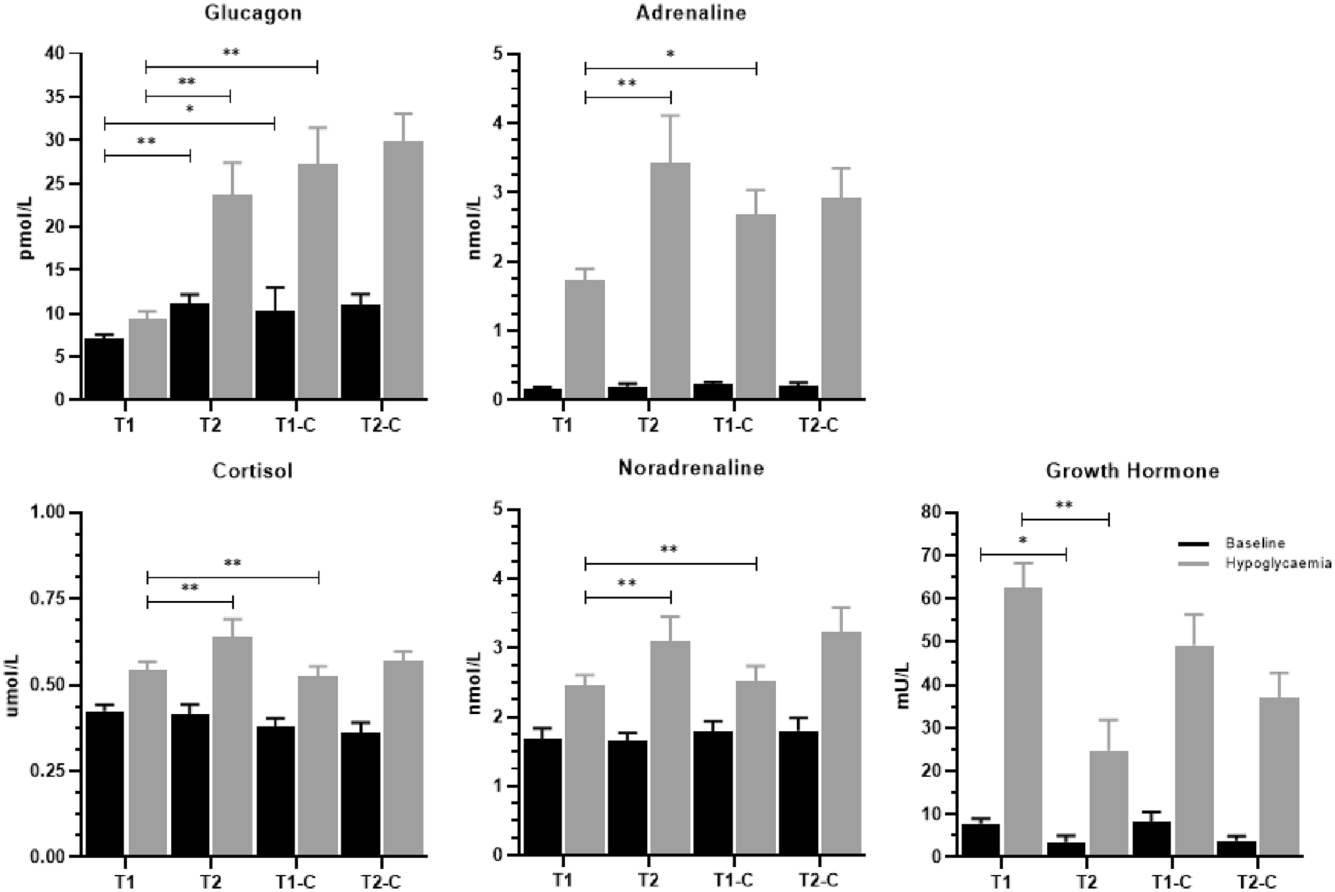
Counterregulatory hormone responses, presented at baseline and hypoglycaemia. Data are shown as means ± SE. T1: Type 1 diabetes, T2: Type 2 diabetes, T1-C: Type 1 controls without diabetes, T2-C: Type 2 controls without diabetes. Response to hypoglycaemia, *p* < 0.05 in all groups. *p < 0.05, **p < 0.001

Glucagon levels were lower in people with type 1 diabetes, as compared to controls without diabetes (7.2 ± 0.4 (7.0 [5.0–9.0]) versus 10.3 ± 1.0 (9.6 [7.0–11.0]) pmol/L,* p* = 0.002) and people with type 2 diabetes (11.2 ± 1.0 pmol/L (12.0 [8.0–14.0]),* p* < 0.001), at the beginning of the euglycaemic phase. There were no significant differences between people with type 2 diabetes and matched control without diabetes (9.2 ± 1.0 (8.5 [7.0–10.0]) pmol/L, *p* = 0.140). The glucagon level increased in response to hypoglycaemia in all groups (*p* ≤ 0.001). However, the response was considerably lower in people with type 1 diabetes (9.4 ± 0.8 (8.0 [7.0–10.0]) pmol/L) as compared to their matched controls without diabetes (30.6 ± 4.7 (25.5 [17.8–35.8]) pmol/L, *p* < 0.001) and in people with type 2 diabetes (23.7 ± 3.7 (18.0 [12.0–28.0]) pmol/L, *p* < 0.001). There were no significant differences between people with type 2 diabetes and matched controls without diabetes (25.2 ± 3.1 (20.5 [18.3–29.9]) pmol/L, *p* = 0.764, Fig. 2 top left panel).

Baseline adrenaline levels did not differ between the subgroups (all *p* > 0.08). In response to hypoglycaemia, adrenaline levels increased in all subgroups but less in people with type 1 diabetes compared to controls without diabetes (1.7 ± 0.2 (1.6 [1.3–5.2]) nmol/L vs 2.7 ± 0.4 (2.8 [1.4–3.9]) nmol/L, *p* = 0.012) and people with type 2 diabetes (3.4 ± 0.7 (2.6 [1.3–5.2]) nmol/L, *p* = 0.001, Fig. 2 top left panel). Baseline noradrenaline and cortisol were comparable in all groups. Both hormones increased in response to hypoglycaemia (*p* ≤ 0.02) in all groups, but the increase in hormone responses was lower in people with type 1 diabetes compared to controls without diabetes (*p* = 0.003) and people with type 2 diabetes (*p* = 0.001). Neither adrenaline or noradrenaline, nor the cortisol responses differed between people with type 2 diabetes and their controls without diabetes (all *p* > 0.4). Growth hormone levels were lower in people with type 2 diabetes than in those with type 1 diabetes, both at baseline (3.4 ± 1.6 vs 7.7 ± 1.3 mU/L, *p* = 0.042) and during hypoglycaemia (24.7 ± 7.1 vs 62.4 ± 5.8 mU/L, *p* = 0.001). Yet, there were no differences when comparing the two diabetes groups with their matched control groups (all *p* > 0.2, Fig. 2). The sensitivity analysis based on propensity score for people with type 1 diabetes and matched controls did not differ for the above findings (ESM, Table 1).

### Symptom scores

**Fig. 3:**
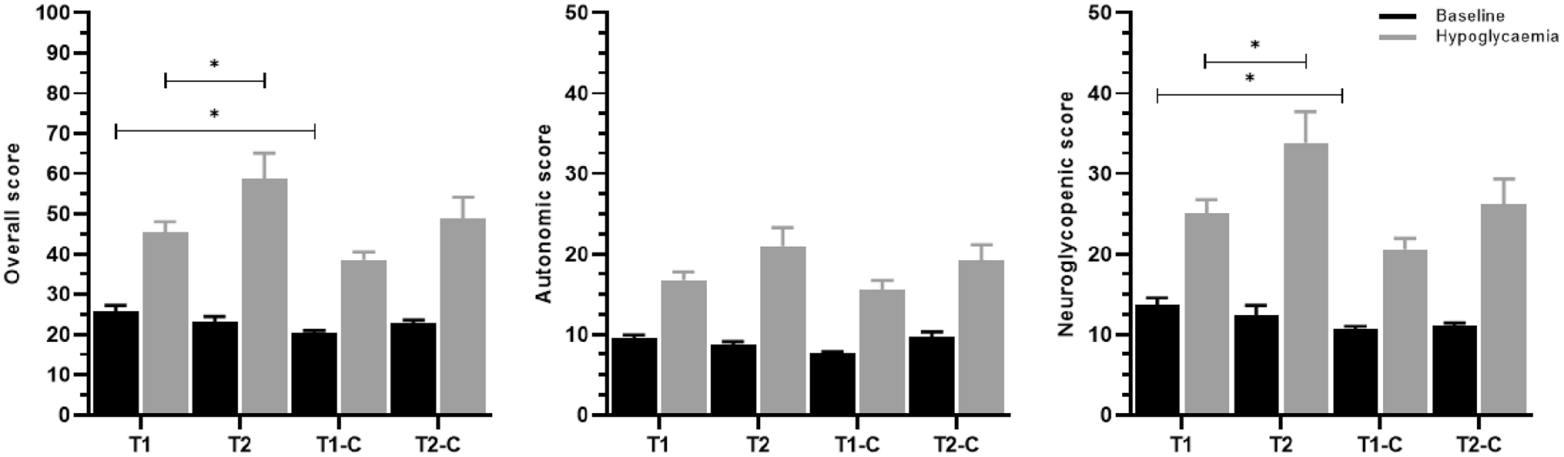
Symptom responses to hypoglycaemia presented at baseline and hypoglycaemia. Data are shown as means ± SE. T1: Type 1 diabetes, T2: Type 2 diabetes, T1-C: Type 1 controls without diabetes, T2-C: Type 2 controls without diabetes. Response to hypoglycaemia, *p* < 0.001 in all groups, *p < 0.05, **p < 0.001

At baseline, symptom scores did not differ between the subgroups, except for a higher overall symptom score in people with type 1 diabetes compared to matched controls without diabetes (25.8 ± 1.4 vs 20.4 ± 0.5, *p* = 0.028), which was driven by a higher neuroglycopenic score (14.9 ± 1.0 vs 10.6 ± 0.4, *p* = 0.016). All groups showed a significant response to hypoglycaemia in overall, autonomic and neuroglycopenic symptom scores (*p* ≤ 0.001)*.* People with type 1 diabetes had a lower overall symptom score in response to hypoglycaemia than people with type 2 diabetes (45.3 ± 2.7 vs. 58.7 ± 6.4, *p* = 0.018), which was also driven by a lower neuroglycopenic score (27.4 ± 1.8 vs. 36.7 ± 4.2, *p* = 0.012). There were no differences in symptom responses between the two diabetes groups compared to their matched control groups (Fig. 3). The matching of people with type 1 diabetes and matched controls based on propensity score did not change these findings (ESM, Table 1).

### Comparison of different insulin infusion rates

When comparing responses to hypoglycaemia using the two different insulin infusion rates of 1.5 and 3.0 mU/kg/min, respectively, in people without diabetes (*n* = 6), neither the hormonal response nor the symptom scores differed between the two conditions (* p* > 0.5).

## Discussion

In this study, in which we directly compared hormonal and symptom responses to hypoglycaemia, we found that people with type 1 diabetes had blunted glucagon and adrenaline responses to hypoglycaemia compared to those with long-standing insulin-treated type 2 diabetes and those without diabetes. Also, the reported symptom responses to hypoglycaemia were lower in people with type 1 diabetes than in people with long-standing insulin-treated type 2 diabetes, but remarkably well-preserved given the blunted counterregulatory hormone responses. Our findings suggest that the counterregulatory deficiencies and reduced symptom responses that are common to type 1 diabetes are not present in people with long-standing insulin-treated type 2 diabetes and preserved beta-cell function.

The blunted glucagon response to hypoglycaemia in people with type 1 diabetes is in line with earlier findings. This defect, which is hypoglycaemia-specific and associated with failing beta cell function, develops in almost all people with type 1 diabetes within 5 years of diagnosis [20, 30–32]. In the people with type 2 diabetes in this study, who despite of more than 13 years diabetes duration and ten years of insulin therapy had preserved endogenous insulin secretion as reflected by measurable C-peptide levels, the glucagon response to hypoglycaemia was in line with that of the control group. This is consistent with previous studies, showing preserved glucagon responses to hypoglycaemia in people in earlier stages of type 2 diabetes [17, 18]. However, our findings contrast with those by Segel et al. [6] who reported a reduced glucagon response in subjects with long-standing insulin-treated type 2 diabetes. This difference may be explained by the participants in that study having more pronounced insulin secretion deficiency with three times lower c-peptide levels compared to our study.

The participants with type 2 diabetes had a preserved adrenaline response, which has been reported by previous studies, including the study by Segel et al. [6]. A potential explanation could be less frequent exposure to hypoglycaemic events than in people with type 1 diabetes [33], which is supported by real-world data [34].

People with type 1 diabetes demonstrated a growth hormone response comparable to that of their matched control group (Fig. 2), which is in line with earlier findings [32, 35–37]. People with type 2 diabetes had a lower growth hormone response to hypoglycaemia than people with type 1 diabetes. Research into the growth hormone response to hypoglycaemia in people with type 2 diabetes is limited. Mumme et al. previously demonstrated an attenuated growth hormone response in people with type 2 diabetes and reasoned that this was due to obesity, which [38] is thought to suppress growth hormone secretion due to elevated free fatty acids [39]. Participants with type 2 diabetes had significantly higher BMI than those with type 1 diabetes, which may explain their lower response. However, as there was no significant difference between the responses of the two control groups, between which there was a greater difference in BMI than between the two diabetes groups, other factors than obesity are likely to be of importance.

Age is reportedly associated with modulation of the counterregulatory hormone and symptom response, resulting in the activation at lower glucose levels in older people without diabetes compared to younger people without diabetes [20, 21]. In this study, the control groups were age-matched with the diabetic groups and should thereby not explain differences between people with diabetes and controls. Likewise, age does not explain the attenuated counterregulatory responses in the group with type 1 diabetes that was younger than the group with type 2 diabetes.

All groups reported significant overall autonomic and neuroglycopenic symptoms in response to hypoglycaemia, with the type 1 diabetes group having a somewhat lower response of overall and neuroglycopenic symptoms than the type 2 diabetes group. The well-preserved symptom response in the type 1 diabetes group relative to their control group was unexpected, and in contrast to the adrenaline response. These findings could indicate that the adrenaline response is not the main driver of the symptom responses [40]. The findings could also, hypothetically be explained by less exposure to hypoglycaemic episodes in people with type 1 diabetes due to improvement of insulin therapy and use of CGM, compared to earlier studies and warrants further investigation.

The glucose nadir in our clamp was chosen to be 2.8 mmol/L to ensure clinically significant hypoglycaemia. This level is in accordance with level 2 hypoglycaemia (< 3.0 mmol/L), as suggested by the International Hypoglycaemia Study Group in their position statement as clinically important hypoglycaemia [41]. The fact that we recorded significant responses of all counterregulatory hormones, as well as symptom responses in all groups, confirms that this level of hypoglycaemia is clinically relevant.

A strength of the current study is the inclusion of a large number of people with type 1 and long-standing insulin-treated type 2 diabetes and two matched control groups without diabetes, studied under the same experimental conditions, which permitted direct comparisons. There are also limitations. These include the one-step design, that precluded us from investigating potential differences in glycaemic thresholds for the responses between the groups. Another limitation is the relative high blood glucose level at the start of the experiment in the group with type 1 diabetes, since a more profound fall in plasma glucose may be expected to trigger counterregulation at higher glucose levels. However, we expect that keeping all participants at a euglycaemic level for 30 min before proceeding to induce hypoglycaemia helped minimizing this risk. Furthermore, the difference in plasma insulin levels between people with type 1 and type 2 diabetes and their control groups during the experiments may be a limitation. However, the crossover sub-study in healthy controls comparing the effect of the two infusion rates resulted in comparable results, suggesting that a two-fold difference in insulin infusion rate cannot explain differences between the two diabetes groups in this study. Although there were no differences in the responses to hypoglycaemia in the sub-study comparing the two different insulin infusion rates, the sub-study was not powered to detect minor differences.

In conclusion, this comparative study showed modest attenuation of glucagon, adrenaline, and symptom responses to hypoglycaemia in people with type 1 diabetes and preserved responses in longstanding insulin-treated type 2 diabetes, possibly as a consequence of preserved beta cell function. In people with type 1 diabetes, hypothetically this may be due to a reduction of the hypoglycaemic burden in recent years by newer insulins and use of insulin pumps and CGM. These findings may explain differences in clinical risk of severe hypoglycaemia between people with type 1 or type 2 diabetes, irrespective of the use of insulin. Understanding diversity and causality in glucose counterregulation in diabetes merits further research.

### Supplementary Information

Below is the link to the electronic supplementary material.

Supplementary file1 (DOCX 519 KB)

## Abbreviations

BMI: Body mass index
CGM: Continuous glucose monitoring
CV: Coefficient of variation
ESM: Electronic supplementary material
GFR: Glomerular filtration rate
GIR: Glucose infusion rate
HbA_1c_: Glycated haemoglobin, fraction_1c_
isCGM: Intermittently scanned continuous glucose monitoring
IQR: Interquartile range
MDI: Multiple daily injections
