# Supplementary material for: Counterregulatory hormone and symptom responses to hypoglycaemia in people with type 1 diabetes, insulin-treated type 2 diabetes or without diabetes: the Hypo-RESOLVE hypoglycaemic clamp study
Source: Acta Diabetol. 2024 Feb 20;61(5):623–33. doi: 10.1007/s00592-024-02239-8 (PMC11055751; doi:10.1007/s00592-024-02239-8)
Supplement: Supplementary file 1 — Supplementary file1 (DOCX 519 KB) [file 592_2024_2239_MOESM1_ESM.docx]

**Electronic supplementary materials (ESM)**

**ESM Methods:**

**Flow chart Blood samples, Hormones – Hypo-RESOLVE**

| **Test** | **Insulin** | **Adrenalin** | **GH**  **Cortisol** | **Glucagon** |
| --- | --- | --- | --- | --- |
| **Material** | Li-Heparin plasma | Li-Heparin plasma | Serum  Serum | EDTA aprotinin plasma |
| **Further instruction** | The glasses must have been on ice for 10 min before sampling | The glasses must have been on ice for 10 min before sampling |  | The glasses must have been on ice for 10 min before sampling |
| **Before centrifugation** | Store on ice until centrifugation | Store on ice until centrifugation |  | Store on ice until centrifugation |
| **Centrifugation** | 1385 RCF in 10 min, 25˚ C | 1385 RCF in 10 min, 25˚ C | 1385 RCF in 10 min, 25˚ C | 1385 RCF in 10 min, 25˚ C |
| **Transfer of supernatant** | All plasma is transferred to Sarsted's tube | All plasma is transferred to a special Sarsted's tube | Distributed in 2 Sarsted tubes | At least 1.2 ml |
| **Pipetting Glass** | 1 x 5 ml Sarstedt tube 13x75mm | 1 x 5 ml Sarsted tube 13x75mm  **Added EGTA etc. (kept in -20 freezer)** | 2 x 5 ml Sarstedt tube 13x75mm | 1 x 5 ml Sarstedt tube 13x75mm |
| **Storage** | **-80 ˚C** | **-80 ˚C** | **-80 ˚C** | **-80 ˚C** |

**Exclusion criteria:**

For all the included people with diabetes, the exclusion criteria were; severe medical or psychological conditions interfering with the perception of hypoglycaemia other than impaired awareness, use of immune-modifying drugs or antibiotics (the past three months), treatment with glucose-modifying (other than insulin, SGLT-2 inhibitors and metformin) agent(s) (e.g. glucocorticoids), use of statins (could be paused two weeks before the experimental day), use of anti-depressive drugs, pregnancy or breastfeeding. Furthermore, people were excluded if they had had any event of cardiovascular disease in the past five years (e.g. myocardial infarction, stroke, heart failure, or symptomatic peripheral arterial disease), auto-inflammatory or auto-immune diseases (other than type 1 diabetes), any infection or vaccination in the past three months, proliferative retinopathy with or without treatment with laser coagulation, diabetic nephropathy (estimated glomerular filtration rate (eGFR) < 60 mL/min), a history of pancreatitis (acute or chronic) or pancreatic cancer. Exclusion criteria for healthy people were the presence of any medical condition that might interfere with the study protocol (e.g. brain injuries, epilepsy, major cardiovascular disease, anxiety disorders, history of pancreatitis etc.), use of medication (except for oral contraceptives, a stable dose of thyroxine or statins (statins could be paused two weeks prior to the experimental day), any infection or vaccination in the past three months.

**ESM Figure 1, Flow chart showing the recruitment process:**


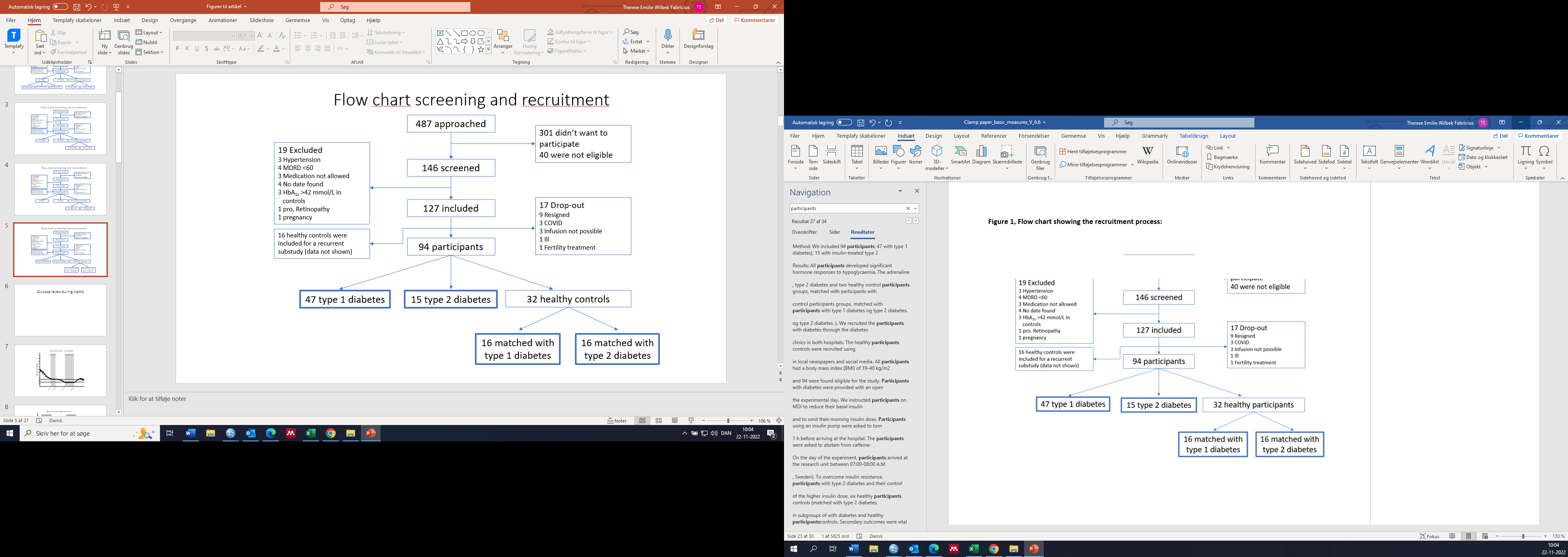


**ESM Table 1: Propensity analysis for Type 1 diabetes and Type 1 controls without diabetes**

|  | **Type 1 diabetes** | **Type 1 controls without diabetes** |
| --- | --- | --- |
| **Baseline measurements** |  |  |
| **Participants, n** | 16 | 16 |
| **Gender (M/F)** | 8/8 | 8/8 |
| **Age, years** | 47.5 [24.3-63.3] | 47.5 [24.5-64.5] |
| **BMI, kg/m^2^** | 24.2 ± 3.1 | 22.6 ± 2.8 |
| **Counterregulatory hormones** |  |  |
| **Glucagon, baseline (pmol/L)** | 6.8 ± 0.7  6.5 [5.0-8.0] | 10.3 ± 1.0  9.5 [7.0-11.0] |
| **Glucagon, hypoglycaemia (pmol/L)** | 7.8 ± 0.8  7.5 [6.0-9.8] | 30.6 ± 4.7**  25.5 [17.8-35.8] |
| **Adrenaline, baseline (nmol/L)** | 0.2 ± 0.03  0.2 [0.1-0.3] | 0.2 ± 0.03  0.2 [0.1-0.3] |
| **Adrenaline, hypoglycaemia (nmol/L)** | 1.4 ± 0.8  1.3 [0.8-1.8] | 2.7 ± 0.4*  2.8 [1.4-3.9] |
| **Cortisol, baseline (umol/L)** | 0.5 ± 0.02 | 0.4 ± 0.03 |
| **Cortisol, hypoglycaemia (umol/L)** | 0.5 ± 0.04 | 0.5 ± 0.03* |
| **Noradrenaline, baseline (nmol/L)** | 1.6 ± 0.2 | 1.8 ± 0.1 |
| **Noradrenaline, hypoglycaemia (nmol/L)** | 2.5 ± 0.3 | 2.5 ± 0.2* |
| **Growth Hormone, baseline (mU/L)** | 11.6 ± 2.8 | 8.4 ± 2.2 |
| **Growth Hormone, hypoglycaemia (mU/L)** | 69.9 ± 11.0 | 49.2 ± 7.0 |
| **Symptoms** |  |  |
| **Overall, baseline** | 24.1 ± 1.5 | 20.4 ± 0.5* |
| **Overall, hypoglycaemia** | 42.6 ± 4.5 | 38.6 ± 1.9 |
| **Autonomic, baseline** | 7.9 ± 0.4 | 7.7 ± 0.2 |
| **Autonomic, hypoglycaemia** | 13.4 ± 1.3 | 15.5 ± 1.2 |
| **Neuroglycopenic, baseline** | 13.7 ± 0.9 | 10.6 ± 0.4* |
| **Neuroglycopenic, hypoglycaemia** | 25.8 ± 3.0 | 20.6 ± 1.4 |

*Baseline data are n (%), mean ± SD or median [IQR]. Counterregulatory hormones are mean± SE or median [IQR]. *p <0.05,**p <0.005,*
